# Identification and validation of reference genes for real-time quantitative RT-PCR analysis in jute

**DOI:** 10.1186/s12867-019-0130-2

**Published:** 2019-04-29

**Authors:** Md. Sabbir Hossain, Rasel Ahmed, Md. Samiul Haque, Md. Monjurul Alam, Md. Shahidul Islam

**Affiliations:** 1 Basic and Applied Research on Jute Project, Bangladesh Jute Research Institute, Dhaka, Bangladesh; 2 Bangladesh Jute Research Institute, Dhaka, Bangladesh

**Keywords:** Reference gene, Gene expression, qRT-PCR, Jute

## Abstract

**Background:**

With the availability of genome sequences, gene expression analysis of jute has drawn considerable attention for understanding the regulatory mechanisms of fiber development and improving fiber quality. Gene expression profiles of a target gene can provide valuable clues towards the understanding of its biological function. Reverse transcription quantitative real-time PCR (qRT-PCR) is the best method for targeted gene expression analysis due to its sensitivity and reproducibility. However, calculating relative expression requires reference genes, which must be stable across various biological conditions. For this purposes, 11 prospective genes namely, 28S RNA, ACT7, CYP, EF1A, EF2, ETIF3E, GAPDH, PP2Ac, PTB, UBC2 and UBI1 were evaluated for their potential use as reference genes in jute.

**Results:**

The expression stabilities of eleven prospective genes were analyzed in various jute plant tissues, such as the root, stick, bark, leaf, flower, seed and fiber, as well as under abiotic (waterlogged, drought and salinity) and biotic stress (infestation with *Macrophomina phaseolina*) conditions with different time points. All 11 genes were variably expressed in different tissues and stress conditions. To find suitable reference genes in different sample sets, a comprehensive approach based on four statistical algorithms such as GeNorm, BestKeeper, NormFinder the ΔCt was used. The PP2Ac and EF2 genes were the most stably expressed across the different tissues. ACT7 and UBC2 were suitable reference genes under drought stress, and CYP and PP2Ac were the most appropriate after inoculation with *Macrophomina phaseolina*. Under salinity stress, PP2Ac and UBC2 were the best genes, and ACT7 and PP2Ac were the most suitable under waterlogged conditions.

**Conclusion:**

Expression stability of reference genes from jute varied in different tissues and selected experimental conditions. Our results provide a valuable resource for the accurate normalization of gene expression experiments in fiber research for important bast fiber crops.

**Electronic supplementary material:**

The online version of this article (10.1186/s12867-019-0130-2) contains supplementary material, which is available to authorized users.

## Background

Gene expression studies have become extremely important in obtaining insights into gene function and understanding molecular mechanisms. Quantitative real-time PCR (qRT-PCR) is considered as the gold standard for the quantification of gene expression due to its specificity, accuracy, sensitivity, reproducibility and wide use in investigating candidate genes [1]. However, the accuracy of qRT-PCR is significantly influenced by RNA integrity, cDNA quality, and the amplification efficiency of qRT-PCR [2]. Among the strategies of normalization of qRT-PCR data to quantify gene expression precisely, normalization with one or multiple reference genes is widely used [3]. While housekeeping genes (HKGs) are commonly used as reference genes for normalization, they vary significantly, depending on the tissue, environmental conditions and species. As a consequence, several studies have been conducted in a number of plants, including flax [4], cannabis [5] cacao [6], cotton [7], soybean [8], and rice [9], to evaluate the expression stabilities of reference genes used for accurate normalization of qRT-PCR analyses.

Compared to all textile fibers except cotton, jute is one of the cheapest and most important lignocellulosic bast fibers. It is gaining popularity due to its biodegradable and renewable characteristics. Global demand for raw jute and jute products is steadily increasing and expected to increase by 200% by 2021 [10]. However, due to the lack of adequate genetic diversity within the available genetic stock [11] the varietal improvement in jute has been impaired. Complete sequencing of the jute genome [12, 13] offers the potential for its use in genome-based precision breeding schemes. However, functional studies involving analyses of gene expression and gene manipulation [14] are required for assigning functions to the gene sequences. In relative gene expression studies, the use of an internal gene reference to normalize test conditions, which must be stable across various biological and ontogenetic conditions, is a prerequisite [15]. In an earlier study, seven HKGs have been tested under different stress conditions in the jute species *Corchorus olitorius* L. [16], although no systematic validation of reference genes has been performed. Moreover, another study was performed on another cultivated jute species, *Corchorus capsularis* L. [17], to identify suitable reference genes (RGs) under different stress conditions and among only three different tissue such as roots, leaves and stems from 15 day old seedlings. These studies were conducted before the availability of genomic data of jute. Here, we report the validation of reference genes to identify the most suitable internal control for normalization of qRT-PCR data from different types of tissue obtained from various organs of jute (*C. olitorius*) in various environmental conditions.

## Methods

### Plant samples

Tossa jute (*C. olitorius* cv. O-4) seeds were sown at the Jute Agricultural Experimental Station in Manikganj, Bangladesh (23.42°N, 89.57°E) during April-September 2017 following recommended cultural practices. Flowers were collected just after blooming and root, stick, bark, leaf and fiber samples were collected from 45-day-old field-grown plants. One-year-old mature seeds were used for RNA extraction. For seedlings, the seeds were grown on filter paper under control conditions (32 °C and 80% humidity on a 13 h light/11 h dark cycle) in a growth chamber and collected after 4 days of germination.

For waterlogged and fungal experiments, seeds were sown in pots containing soil under a controlled environment (32 °C and 80% humidity on a 13 h light/11 h dark cycle) in a greenhouse and grown for 3 weeks after germination (at the 4–6 leaf stage). For waterlogged stress, pots containing seedlings were placed into a tank that was submerged under water at 2 cm above the soil surface. Stressed seedlings were then harvested at 0, 2, 4, 8, 12 and 24 h after treatment. For fungal stress, the seedlings were sprayed with a suspension containing 10^8^ inocula/ml *Macrophomina phaseolina* and immediately transferred into a moist chamber (100% humidity; required for optimal infection). Sprayed seedlings were then collected at 0, 4, 8, 12, 24 and 48 h after treatment for gene expression analysis. For drought and salt stress, the seedlings were grown in 1/3rd strength Hoagland’s solution in a greenhouse. Three-week-old seedlings (at the 4–6 leaf stage) were subjected to 30% PEG [18] and 300 mM NaCl [19] to simulate drought and salinity stress, respectively. After 0, 2, 4, 8, 12 and 24 h of treatment, seedlings were collected for RNA extraction which allows comparing the expression levels between time points. The early sampling (0–4 h) consists the rapid induction of signal transduction components and late sampling (4-24 h) consists metabolic adaptation [20]. A total of 32 samples from four different stress conditions at various time points including eight tissue-specific samples were carefully harvested, immediately frozen in liquid nitrogen and stored at − 80 °C until use. Three biological experimental replicates were collected for each type of different tissue and stress treatment on different dates.

### Total RNA extraction and cDNA synthesis

One gram of tissue samples was disrupted in liquid nitrogen to a fine powder using a mortar and pestle. Total RNA was extracted from all samples (except fiber and 4-day-old seedling samples) using an in-house modified Cetyltrimethylammonium bromide (CTAB) protocol (unpublished results). For fiber samples, total RNA was extracted as described by Islam et al. [12], and for 4-day-old seedlings a Guanidinium thiocyanate (GNTC) based protocol was used [21]. Total RNA concentration and purity were determined using a NanoDrop 2000 spectrophotometer (NanoDrop, ThermoScientific, USA). Total RNA integrity was evaluated by 1% agarose gel electrophoresis. The absence of genomic DNA in the sample was confirmed by PCR using crude RNA without reverse transcription as a template and assessed by 1.2% agarose gel electrophoresis. Before cDNA synthesis, total RNA was treated with amplification grade DNase I (SigmaAldrich, Germany) to remove any traces of genomic DNA according to the manufacturer’s instructions. Subsequently, the first strand complementary DNA (cDNA) was synthesized from 2 µg of total RNA using the RevertAid First Strand cDNA Synthesis Kit (Thermo Fisher Scientific, USA) according to the manufacturer’s instructions. Following these steps, the samples were incubated with RNaseH (Thermo Fisher Scientific, USA) to degrade the RNA strand of any RNA–DNA hybrids according to the manufacturer’s instructions. The cDNA was then stored at − 20 °C until use.

### Primer design and amplification efficiency

A total of 11 candidate genes based on previous studies on different plants, including jute [16, 17], flax [4], cotton [7], soybean [8], and rice [9], were used to identify the most suitable genes for gene expression analysis using qRT-PCR. The selected candidate genes were 28S ribosomal RNA (28S), actin 7 (ACT7), cyclophilin (CYP), elongation factor 1-α (EF1A), elongation factor 2 (EF2), eukaryotic translation initiation factor 3E (ETIF3E), glyceraldehyde 3-phosphate dehydrogenase (GAPDH), the catalytic subunit of protein phosphatase 2A (PP2Ac), polypyrimidine tract-binding protein homolog (PTB), ubiquitin-conjugating enzyme E2 (UBC2) and ubiquitin 1 (UBI1). For our study, the sequences of the candidate genes were retrieved from the TAIR database (www.arabidopsis.org), and potential homologs of these genes were identified in the *C*. *olitorius* jute genome and transcriptome data [12] using BLASTN. Primer pairs for the candidate genes were designed using online tools from IDT (sg.idtdna.com) and GenScript (www.genscript.com). Attentions have been given to span exon–exon junction while primers were designed. The specificity of these primer pairs were evaluated by PCR followed by 1.2% agarose gel electrophoresis and melt curve analysis during qRT-PCR. Calculation of the amplification efficiency was determined from the slope of the standard curve. Standard curves were generated by performing qRT-PCR on each gene using serially diluted cDNA samples. The amplification efficiency was then calculated using the formula E = (10^−1/slope^ − 1) × 100%.

### Reverse transcription quantitative real-time PCR

qRT-PCR was carried out in a 96-well plate on a Roche LightCycler^®^ 480 instrument II using LightCycler^®^480 SYBR Green I Master mix (Roche Diagnostics, Germany). The qRT-PCR mixture contained 10 µl of 2X SYBR Green I Master mix, 10 ng of cDNA, 0.4 µM of each primer and PCR-grade water up to total volume of 20 µl. Thermal cycling was composed of an initial denaturation step at 95 °C for 5 min followed by 40 cycles of denaturation at 95 °C for 10 s, primer annealing (Additional file 1: Table S1) for 10 s and extension at 72 °C for 30 s. Afterwards, the dissociation curve was obtained by melting the amplicon from 60 to 95 °C. All qRT-PCR reactions were performed in triplicates with a negative control (no template) and repeated three times on three biological replicates.

### Data analysis

GeNorm, NormFinder, BestKeeper and a standard comparative method (ΔCt) were used to evaluate the expression stability of the potential candidate genes. In GeNorm and NormFinder, the raw Ct values were converted into relative expression levels using the formula 2^−∆Ct^, where the lowest Ct sample was used as a calibrator (∆Ct = each corresponding Ct value − minimum Ct value). These converted Ct values were used in analysis [3, 22]. Raw Ct values were used for BestKeeper and ∆Ct analyses [23, 24]. GeNorm determines the most stable genes based on the average pairwise variation of each gene compared to that of other genes and calculates the gene expression stability (M), where the lowest M value is considered the most stable gene. In addition, it determines the optimal number of internal control genes required for qRT-PCR normalization by performing stepwise exclusions of the least stable gene. A pairwise variation cut-off value (V_n_/V_n+1_) below 0.15 indicates that an additional internal gene is not required for qRT-PCR normalization [3]. NormFinder calculates the most reliable gene based on intra-and intergroup variations of a sample set, while BestKeeper determines suitable RGs according to a coefficient of correlation between candidate genes [22, 23]. The ∆Ct approach evaluates the best endogenous gene by comparing the relative expression of each gene with that of other genes [24]. As different algorithms show different rank according to their analytical principles, a comprehensive ranking was generated based on the geometric mean of the individual ranking values obtained from the four algorithms to better evaluate the candidate genes used in this study. To validate the selected reference genes for qRT-PCR normalization, the expression patterns of the ethylene-responsive transcription factor (ERF7A) and the hypoxia-responsive/ethylene-responsive transcription factor (ERF7B) were analyzed using the most and least stable genes according to the 2^−∆∆Ct^ formula described by Livak et al. [25].

## Results

### Primer specificity, efficiency and expression profiling of candidate reference genes

**Fig. 1:**
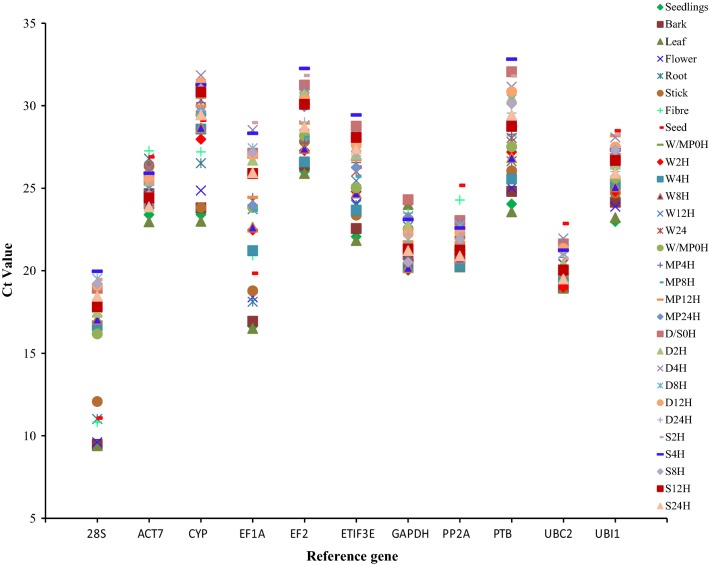
Expression profiles of candidate reference genes across all the experimental samples. The expression profiles of the 11 candidate reference genes in absolute Ct values over different samples including tissues samples, waterlogged stress samples, fungal stress samples, drought stress samples and salinity stress samples

A total of 11 candidate jute housekeeping genes (HKGs), including 28S, ACT7, CYP, EF1A, EF2, ETIF3E, GAPDH, PP2Ac, PTB, UBC2 and UBI1, were targeted to select suitable internal controls for gene expression studies using qRT-PCR (Additional file 1: Table S1). Additionally, ERF7A and ERF7B were used to further validate the accuracy of the identified internal controls under waterlogged stress in jute. The specificity of the primer pairs was confirmed via gel electrophoresis and dissociation curve analysis. The presence of a single band with the expected amplicon size (Additional file 2: Figure S1) and a single peak in the dissociation curve (Additional file 2: Figure S2) suggested that the primers amplified specific products. The efficiency of the primers ranged from 90.86 to 113.07% (Additional file 1: Table S1), and the regression coefficient was > 0.98 (Additional file 2: Figure S3). The quantification cycle (Cq) values obtained by qRT-PCR were used to provide an overview of the expression levels of the candidate genes across all the samples. The mean Cq values of the reference genes ranged from 16 to 29 for different samples as shown in Fig. 1. The 28S gene was found to be the most abundantly expressed with the lowest mean Cq value (16.12 ± 3.17), followed by UBC2 (20.52 ± 1.15), while EF2 was expressed at the lowest level (29.02 ± 2.02). The other internal genes used in the experiment were moderately expressed, with mean Cq values ranging from 21 to 28. Additionally, ACT7 showed the least variation in its transcript level with a coefficient variation (CV) of 4.87%, across all samples and UBC2 showed the second least variation in gene expression with a CV of 5.60%, followed by PP2Ac (CV = 5.94%). Whereas 28S was the most variable reference gene (CV = 23.01%) followed by EF1A (CV = 16.07%). The CVs of the other reference genes among all samples ranged from 6.3 to 9.0%. These results clearly indicate that the expression of internal control genes varied, and validating reference genes for use in qRT-PCR normalization is therefore necessary.

### Analysis of gene expression stability

**Fig. 2:**
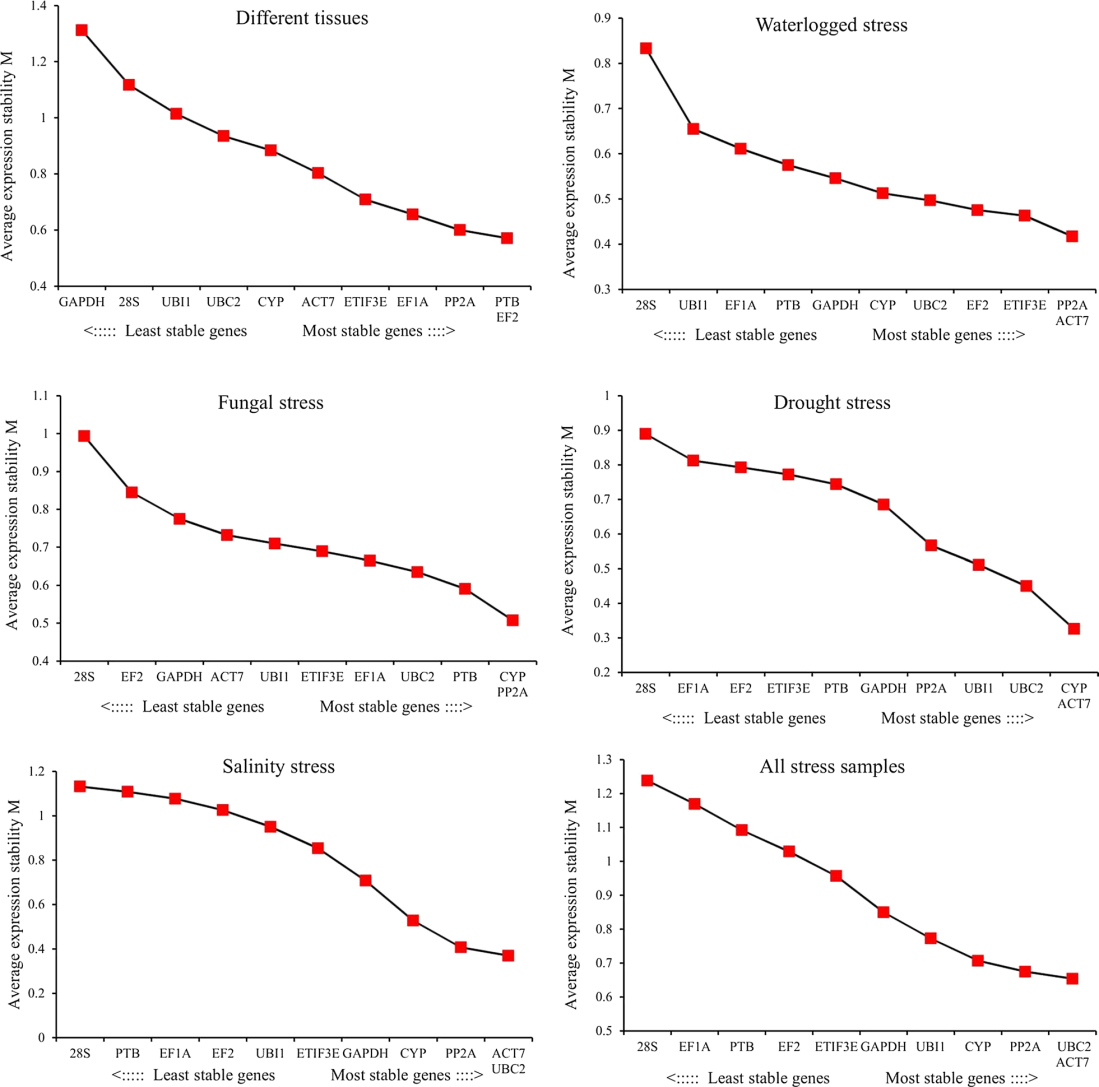
Average expression stability values (M) of 11 candidate reference genes in jute as calculated by GeNorm. M values of the reference genes were calculated by GeNorm algorithm for different tissues samples, waterlogged stress samples, fungal stress samples, drought stress samples, salinity stress samples and all stress samples. The lowest average expression stability (M) value indicates more stable gene

**Fig. 3:**
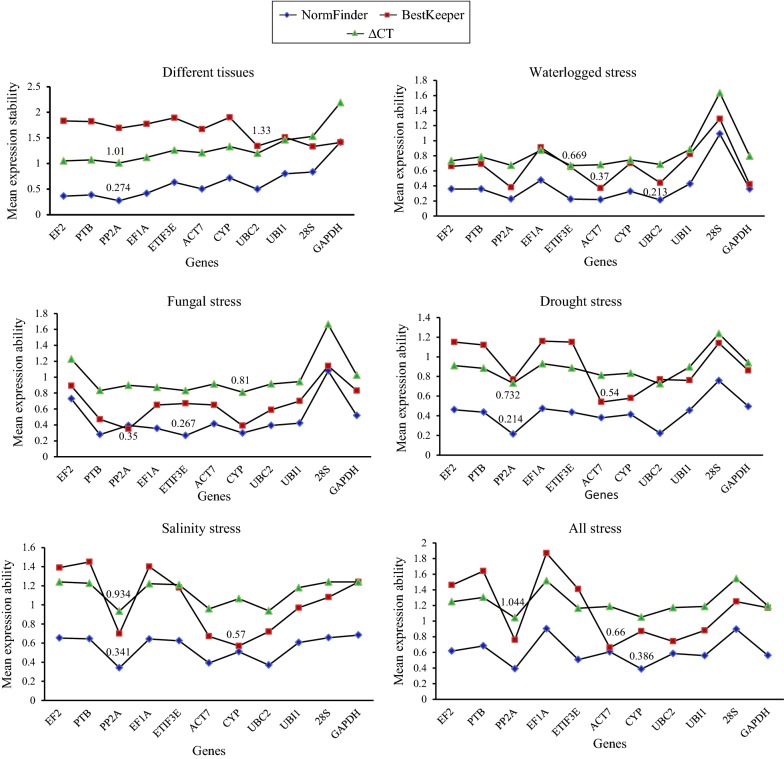
Gene expression stability values of the candidate reference genes calculated by NormFinder, BestKeeper and ΔCt. Stability value of the reference genes were calculated for different tissues samples, waterlogged stress samples, fungal stress samples, drought stress samples, salinity stress samples and all stress samples using NormFinder, BestKeeper and Δct. The lowest stability value indicates most stable expression

GeNorm analysis of different parts of jute revealed that EF2 and PTB were the best pair of stable genes with a mean expression stability (M) value of 0.571, followed by PP2Ac (M = 0.60) and EF1A (M = 0.656), as shown in Fig. 2. NormFinder identified that PP2Ac (SV = 0.274) has shown the most stable gene for normalizing qRT-PCR data (Fig. 3). This gene also showed the most stable expression according to ΔCt analysis, with an SD value of 1.01. Based on the NormFinder analysis, EF2 (SV = 0.363) and PTB (SV = 0.386) were the second and third most stably expressed, respectively. According to the results of BestKeeper analysis, 28S (SD = 1.33) was found to be the most suitable gene, followed by UBC2 (SD = 1.34) and GAPDH (SD = 1.41). Considering the ΔCt analysis, EF2 (SD = 1.05) and PTB (SD = 1.07) were the second and third most stable genes, respectively. GAPDH was the least stable gene for qRT-PCR normalization by all the algorithms used in the study with the exception of BestKeeper. BestKeeper found CYP to be the worst internal gene when considering gene expression analyses in different parts of jute.

For waterlogged conditions, GeNorm indicated that the most stable pair of genes was ACT7 and PP2Ac with an M value of 0.417 (Fig. 2). Likewise, according to BestKeeper analysis, ACT7 (SD = 0.37) and PP2Ac (SD = 0.38) were ranked as the first and second most stable genes, respectively (Fig. 3). The NormFinder algorithm results differed slightly from those of GeNorm and BestKeeper. NormFinder selected UBC2 (SV = 0.213) as the most suitable gene, followed by ACT7 (SV = 0.218) and ETIF3E (SV = 0.224). However, ETIF3E (SD = 0.669) showed the least variation in expression by ΔCt analysis, while PP2Ac (SD = 0.674) and ACT7 (SD = 0.681) were the second and third least variable, respectively. Based on all the algorithms, 28S showed the most variability among all the genes with low stability values. UBI1 and EF1A were ranked at either the 10^th^ or 9^th^ positions according to the four algorithms under waterlogged conditions.

For seedlings inoculated with fungus, PP2Ac displayed the best stability performance with an SD value of 0.35 followed by CYP (SD = 0.39) and PTB (SD = 0.47) as reported by BestKeeper (Fig. 3). Similarly, CYP (M = 0.507) was the most appropriate RG along with PP2Ac (M = 0.507) based on the GeNorm analysis (Fig. 2). ETIF3E (SV = 0.267) and PTB (SV = 0.279) were selected as the two most stable internal genes using NormFinder, while CYP and ETIF3E appeared to be the two best RGs with SD values of 0.81 and 0.831, respectively, when evaluated by ΔCt analysis. Similar to the waterlogged conditions, 28S exhibited the most variation under fungal stress conditions according to all the algorithms, followed by EF2.

Under drought condition, the results of BestKeeper were similar to those of the GeNorm ranking. Both methods ranked ACT7 and CYP as the top two RGs for normalization (Figs. 2, 3). However, UBC2 appeared as the third most stable gene by GeNorm, while UBI1 was the third most stably expressed according to BestKeeper. In the NormFinder analysis, PP2Ac (SV = 0.214) and UBC2 (SV = 0.223) proved to be the best candidate genes, followed by ACT7 and CYP with stability values of 0.380 and 0.413, respectively. Likewise, the top four genes according to the NormFinder analysis were also the top four candidate genes in the ΔCt analysis. The stability values of UBC2 and PP2Ac were 0.726 and 0.732, respectively, indicating that UBC2 and PP2Ac had the least variation in gene expression. With the exception of the BestKeeper algorithm, 28S was found to be the most unstable during drought conditions according to all the methods. EF1A, on the other hand, shown the worst according to the BestKeeper algorithm, followed by ETIF3E. GAPDH was ranked as the second most variable gene when evaluated by ΔCT and NormFinder, whereas EF1A was identified to have the second worst gene expression according to GeNorm.

In salinity conditions, the four candidate genes ACT7, UBC2, PP2Ac and CYP emerged as the top four suitable genes according to all the algorithms, although their ranking orders differed, as presented in Figs. 2 and 3. The results of GeNorm (Fig. 2) revealed that ACT7 and UBC2 (M = 0.37) were the best pair of stable genes, followed by PP2Ac (M = 0.407) and CYP (M = 0.528). The candidate genes were ranked by NormFinder as PP2Ac, UBC2, ACT7 and CYP with stability values of 0.341, 0.370, 0.392 and 0.510, respectively. According to BestKeeper, CYP (SD = 0.57) was classified as the most suitable gene for accurate normalization, followed by ACT7 (SD = 0.67), while PP2Ac (SD = 0.70) and UBC2 (SD = 0.72) were the third and fourth most suitable, respectively. PP2Ac (SD = 0.934) and UBC2 (SD = 0.938) were found to be the best internal genes, whereas ACT7 (SD = 0.959) and CYP (SD = 1.065) ranked third and fourth, respectively, when evaluated by ΔCt. 28S showed variable expression under salinity stress according to GeNorm and ΔCt. GeNorm found PTB to be the second worst gene, while GAPDH was the second least stable gene according to ΔCt. In BestKeeper analysis, PTB was the most variable gene, followed by EF1A. By contrast, GAPDH and 28S were the worst scoring genes based on NormFinder.

Considering all stress samples, the rankings of the 11 candidate RGs based on different algorithms for qRT-PCR normalization are presented in Figs. 2 and 3. ACT7 and UBC2 were considered the best pair of reference genes (Fig. 2) with an M value of 0.654, followed by PP2Ac (M = 0.675). By contrast, 28S and EF1A were the least consistently expressed according to GeNorm, with the highest M values of 1.23 and 1.16, respectively. Likewise, BestKeeper ranked ACT7 and UBC2 as the top two internal genes with SV values of 0.66 and 0.74, respectively, whereas EF1A (SV = 1.87) and PTB (SV = 1.64) were found to be the most unstable genes for normalization. CYP and PP2Ac emerged as the most appropriate RGs with SVs of 0.386 and 0.390, respectively, while EF1A (SV = 0.902) and 28S (SV = 0.896) showed the most variation according to NormFinder. The ranking order of the ΔCT analysis was marginally different from NormFinder. PP2Ac, CYP and ETIF3E were identified as the three best endogenous control genes with SD values of 1.044, 1.051 and 1.164, respectively. By contrast, 28S (SD = 1.544) and EF1A (SD = 1.517) were found to be the least stable genes for normalization in all stress samples.

### Determination of the optimal number of reference genes

**Fig. 4:**
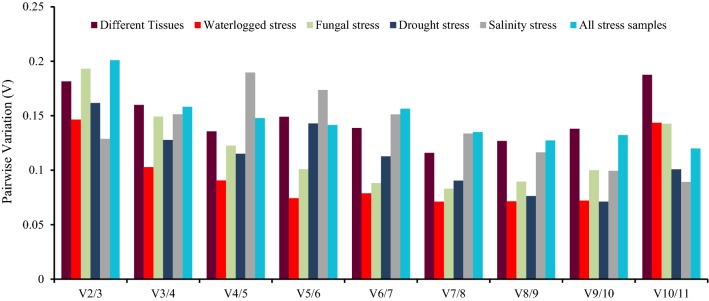
Determination of the optimal number of internal genes for accurate normalization. The pairwise variation (V = V_n_/V_n+1_) was calculated between normalization factor NF_n_ and NF_n+1_ was performed by GeNorm to determine the optimal number of reference genes required for qRT-PCR normalization in all the six sample sets. Values under 0.15 indicate that no additional genes are required for the normalization

The optimal number of RGs required for accurate normalization to obtain precise qRT-PCR results was also determined by pairwise variation (V_n_/V_n+1_) using GeNorm. According to Vandesompele et al. [3], a cut-off value of V_n_/V_n+1_ < 0.15 suggests that the addition of another reference gene would have no significant contribution to normalization in qRT-PCR analysis. As shown in Fig. 4, the pairwise variations V_2/3_ (0.181) and V_3/4_ (0.159) were higher than 0.15, while the V_4/5_ variation was 0.135. Therefore, the combination of four genes should be used to normalize gene expression for different parts of jute. Under waterlogged and salinity conditions, the V_2/3_ was lower than 0.15 which indicates that two internal genes would be sufficient (Fig. 4). On the other hand, the inclusion of a third reference gene would be useful for transcript normalization in qRT-PCR under fungal and drought stress, as the V_3/4_ values in these conditions were 0.149 and 0.127, respectively (Fig. 4). While combining all of stress samples, the combination of four genes should be used to execute accurate qRT-PCR normalization, as the V_4/5_ was lower than 0.15 (Fig. 4).

### Comprehensive stability rankings

**Fig. 5:**
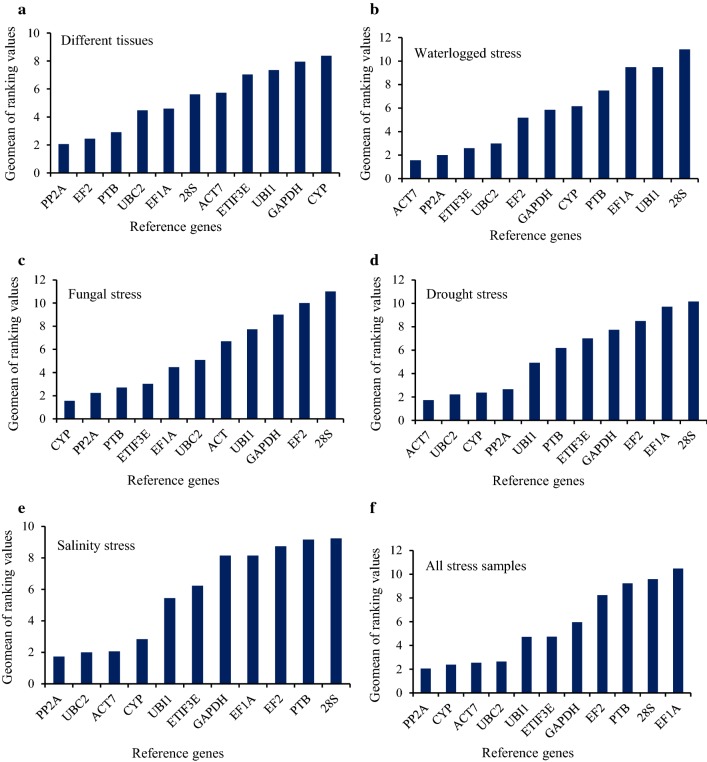
Comprehensive ranking of the candidate reference genes for the six sample sets. Geometric means of ranking values obtained from different algorithms were used to compute the comprehensive stability ranking for **a** different tissues namely, root, stick, bark, leaf, flower, seed, seedlings and fiber, **b** waterlogged stress, **c** fungal stress, **d** drought stress, **e** salinity stress and **f** all stress samples

As different algorithms gave different ranking orders according to their strategies, it was difficult to recognize the best reference gene. For example, UBC2 appeared as the most stable gene according to NormFinder, while it was ranked as fifth by GeNorm under waterlogged conditions (Fig. 3). Therefore, a comprehensive approach considering all four algorithms was performed to better evaluate the candidate reference genes. PP2Ac and EF2 were ranked as the top two reference genes for normalization based on comprehensive ranking, followed by PTB and UBC2, as shown in Fig. 5a. The least stable genes were CYP and GAPDH when different parts of jute were considered. ACT7 was the best reference gene for both waterlogged and drought conditions in qRT-PCR normalization, while PP2Ac and UBC2 ranked as second for waterlogged and drought stresses, respectively (Fig. 5b, d). For fungal stress, CYP was classified as the most appropriate internal gene, whereas PP2Ac was the best gene under salinity stress conditions (Fig. 5c, e). 28S was found to be the worst reference gene for qRT-PCR normalization under all stress conditions. In the case of all stress sample sets, PP2Ac ranked as the best RG, followed by CYP and ACT7, while EF1A and 28S were the two worst internal genes for accurate normalization (Fig. 5f).

### Reference gene validation

**Fig. 6:**
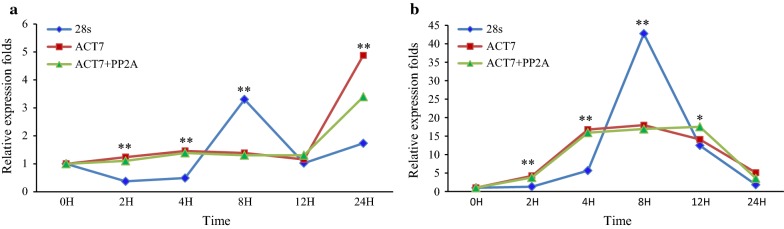
Validation of selected reference genes under waterlogged conditions. Relative expression of **a** ERF7A and **b** ERF7B using selected reference genes, including the most and least stable genes for normalization under waterlogged conditions after 0, 2, 4, 8, 12 and 24 h. Bars indicate the standard error calculated from replicates. Single asterisk indicates statistically significant (p < 0.05); double asterisk indicates greatly statistically significant (p < 0.01)

To validate the effectiveness of the selected internal genes, the expression patterns of two genes, ERF7A and ERF7B (homologous to *Arabidopsis* RAP2.3 (At3g16770) and HRE2 (At2g47520), respectively [26]) were analyzed under waterlogged conditions. The relative transcript abundances of these genes were normalized to those of the most and least stable genes (ACT7 and 28S) and a combination of the two most genes (ACT + PP2Ac). The expression of ERF7A was slightly increased under waterlogged conditions at 12 h and up regulated by 4.87- and 3.39-fold at 24 h when only ACT7 and ACT7 + PP2Ac were used as normalization factors, respectively (Fig. 6a). On the other hand, the relative expression of ERF7A displayed strong fluctuations with different up/down regulated fold changes when 28S was used for transcript normalization. The transcript level of ERF7A decreased at 2–4 h, then increased rapidly and peaked at 8 h. Thereafter, it decreased at 12 h and increased again at 24 h. This expression pattern differed with normalization by ACT and ACT7 + PP2Ac (Fig. 6a). Larger discrepancies were also observed for the expression of ERF7B among the most and least stable reference genes. The relative expression of ERF7B increased at 2 h, followed by a sharp accumulation at 4 h, an increasing trend at 8 h, down regulation at 12 h and a decreasing accumulation at 24 h when normalized by ACT and ACT7 + PP2Ac (Fig. 6b). By contrast, when 28S was used, the expression of ERF7B gradually increased at 4 h, increased to its highest level at 8 h, rapidly decreased at 12 h, and then continued a decreasing trend at 24 h (Fig. 6b).

## Discussion

Fiber is the ultimate product of jute, and identifying key genes in fiber cell biogenesis is crucial for developing new genetic variability with better fiber quality. Therefore, gene expression profiling among different tissues can help to understand the genetic basis of fiber cell biogenesis in jute. The results of the present study describes a systematic approach to identify suitable reference genes for the accurate normalization of gene expression analysis using qRT-PCR across different tissues and under various stress conditions in *C. olitorius*. A comprehensive ranking based on four algorithms was used to select the appropriate internal genes among different jute tissues and under various stress conditions for accurate qRT-PCR normalization. Two studies have been conducted earlier *C. olitorius* [16] and *C. capsularis* [17] to obtain appropriate RGs. In case of *C. olitorius* young seedlings (4 days old) were used with different stress conditions and no tissue samples were considered [16]. On the other hand, the study on *C. capsularis* considered three tissue types (roots, leaves and stems) of 15-day-old seedlings and three statistical programs were used to select stable genes [17]. In addition, no reference gene stability study has been reported earlier under waterlogged conditions for jute species.

Based on our comprehensive ranking system, PP2Ac and EF2 were the two most appropriate internal genes for different jute tissues. Similar results were also found in cotton for different plant organs [7]. Previously, TUB and UBI were identified as the most appropriate RGs in *C. capsularis* [17]. However, UBI showed variable expression across the different tissues analyzed in the present study. Different species or ages of plants might be the reason underlying the differing stability rankings of RGs. Since jute plant growth is relatively rapid between 30 and 60 days after germination, we collected root, bark, stick, leaf and fiber from 45-day-old plants to better survey the genes associated with fiber cell development. Under waterlogged conditions, ACT7 and PP2Ac were selected as suitable reference genes for accurate normalization according to our comprehensive ranking. ACT and PP2Ac were the 1st and 3rd most suitable genes in a waterlogged stress environment for *Vigna angularis* [27] which is consistent with our results. Our results were also in accordance *Oenanthe javanica* species, in which ACT and PP2Ac showed steady expression under abiotic stress [28].

We observed that CYP, PP2Ac and PTB were the top three ranked internal genes during interaction with *M. phaseolina*, a devastating necrotrophic fungus that infects over 500 plants [29]. Our results were also corroborated by the results of other studies in which CYP was reported to be the best reference gene in grapevine *Phaeomoniella chlamydospora* interactions [30], in cabbage infected with fungi [31] and in soybeans infected with *M. diffusa* [32] but was found to be inappropriate for *Vigna mungo* [33]. On the other hand, PP2Ac has been demonstrated to be a good internal gene in *Actinidia deliciosa* leaves infected with *Pseudomonas* [34] and in virus-infected *Nicotiana benthamiana* [35]. Under drought conditions, ACT7 and UBC2 showed stable expression in jute. In other studies, UBC was also found to be one of the most appropriate internal genes in jute under drought stress, but ACT showed poor performance [16, 17]. However, ACT was shown to be a good choice in carrots [36] and *O. javanica* [28] which supports our findings.

Under salinity stress conditions, PP2Ac was the strongest performer, followed by UBC2 and ACT7. Similarly, PP2Ac is reported stably expressed under salt-treated *Brassica napus* [37] and creeping bentgrass [38]. UBC2 has also been noted to be a reliable internal gene in jute [16], *Salicornia europaea* [39], pigeon pea [40] and *Stipa grandis* [41]. ACT7 was reported to be an appropriate reference gene for jute [17], *Salix psammophila* [42] and creeping bentgrass [38] under salinity stress environment. However, the results of our study differed from those of previous studies on jute housekeeping genes, as they reported that EF1A was one of the most stable genes [16, 17], while it was ranked as 9th in our study. PP2Ac, CYP, and ACT7 were identified as reliable RGs for qRT-PCR normalization in jute when all stress samples were considered. Consistent with our results, PP2Ac is reported as a stable internal gene in sorghum [43] and *Caragana intermedia* [44] under various abiotic stress conditions. Additionally, CYP was identified as a suitable endogenous control for qRT-PCR in sorghum [43] and maize [45] under multiple stress conditions. Moreover, ACT7 has been noted to have stable expression under different abiotic stress conditions in carrots [36] and *Oenanthe javanica* [28].

To assess the suitability of the identified reference genes, we analyzed the expression patterns of two ERF7 genes, ERF7A and ERF7B, which are homologs to the RAP2.3 and HRE2 genes in Arabidopsis, respectively. Under waterlogged conditions, the transcript level of ERF7A was nearly unchanged after 24 h of treatment when the best ranked gene and a combination of the two top ranked genes were used as normalization factors. A similar expression pattern was observed under waterlogged stress in Arabidopsis [26]. In contrast, variable expression patterns were found for ERF7A when the lowest ranked gene was used for qRT-PCR normalization. In addition, the expression of ERF7B was up regulated under waterlogged conditions upon normalization to the top ranked genes, which was also observed in *Arabidopsis* [26]. Larger discrepancies in the expression of ERF7B were observed when the worst gene was used for qRT-PCR normalization. These results suggest that the identified genes are suitable for transcript normalization in *C. olitorius*. Therefore, the results of the study indicate that the selection of appropriate internal genes as normalization factors is crucial for the quantification of target gene expression using qRT-PCR.

The expression stabilities of the 11 candidate HKGs were analyzed using four different statistical algorithms, and their results were used together to obtain a comprehensive ranking based on the geometric means. We recommend that the PP2Ac and EF2 genes are suitable reference genes across different tissues and ACT7 and PP2Ac genes are suitable for waterlogged conditions. For gene expression studies under drought stress, ACT7 and UBC2 are the best genes, and CYP and PP2Ac are suitable genes for *M. phaseolina* stress. PP2Ac and UBC2 proved to be the most stable internal controls under salinity stress conditions. These results will facilitate accurate gene expression quantification among different tissues and multiple stress conditions in jute.

## Conclusion

In this study, we have tested the expression stabilities of eleven candidate genes in eight tissue samples from jute plants and four experimental conditions (drought stress, waterlogged condition, salinity and fungal infection) with four statistical algorithms, GeNorm, NormFinder, BestKeeper and ΔCt method. Additionally, the expression pattern of target gene ERF7A and ERF7B was determined under waterlogged condition to further verify the reliability of the identified stable reference genes. Here we identified that PP2Ac and EF2 genes are the most stably expressed across different tissues. While expression of ACT7 and UBC2 were most stable under drought stress and CYP and PP2Ac were the most appropriate reference genes after inoculation with *M. phaseolina*. For gene expression under salinity stress, PP2Ac and UBC2 were the best genes and ACT7 and PP2Ac were the most suitable under waterlogged conditions. The findings presented in this study form a highly useful platform for future transcriptional analysis in jute.

## Additional files

**Additional file 1:** Supplementary Table.

**Additional file 2:** Supplementary Figure.

## Abbreviations

qRT-PCR: quantitative real-time PCR
HKGs: housekeeping genes
RGs: reference genes
CTAB: cetyltrimethylammonium bromide
GNTC: guanidiniumthiocyanate
cDNA: complementary DNA
28S: 28S ribosomal RNA
ACT7: actin7
CYP: cyclophilin
EF1A: elongation factor 1-α
EF2: elongation factor 2
ETIF3E: eukaryotic translation initiation factor 3E
GAPDH: glyceraldehyde 3-phosphate dehydrogenase
PP2Ac: catalytic subunit of protein phosphatase 2A
PTB: polypyrimidine tract-binding protein homolog
UBC2: ubiquitin-conjugating enzyme E2
UBI1: ubiquitin 1
ΔCt: comparative method
ERF7A: ethylene-responsive transcription factor
ERF7B: hypoxia-responsive/ethylene-responsive transcription factor
Cq: quantification cycle
CV: coefficient variation
SV: stability value
SD: standard deviation
