# Supplementary material for: Identification and validation of reference genes for real-time quantitative RT-PCR analysis in jute
Source: BMC Mol Biol. 2019 Apr 29;20:13. doi: 10.1186/s12867-019-0130-2 (PMC6489354; doi:10.1186/s12867-019-0130-2)
Supplement: Supplementary file 1 — Additional file 1: Supplementary Table. [file 12867_2019_130_MOESM1_ESM.docx]

**Table S1.** Candidate reference genes and target genes primer sequences, amplicon length and qRT-PCR analysis

| Gene Symbol | Gene Descripton | Gene_ID | Forward Primer (5'–3’) | Reverse Primer (5'–3’) | Amplicon Length (bp) | Annealing Tm° C | PCR efficiency | R^2^ |
| --- | --- | --- | --- | --- | --- | --- | --- | --- |
| 28S | 28S Ribosomal RNA | COLO4_rRNA_04084 | TGA AGA AAT TCA ACC AAG CG | GAC AGG GAC AGT GGG AAT CT | 127 | 48 | 105.35% | 0.996885 |
| ACT7 | Actin 7 | COLO4_04861 | CTG TTG GTG AGG CAT TGT TC | GCT TGC TTC TTT CTG GAA CC | 188 | 50 | 90.86% | 0.998264 |
| CYP | Cyclophilin | COLO4_17310 | TTG ATA TCG ATG GTC AGC GT | TCC CTT ATA GTG CAG GGC TT | 119 | 50 | 92.71% | 0.994866 |
| EF1A | Elongation Factor 1-α | COLO4_27068 | TCC AAG GCA AGG TAT GAT GA | TTG GAC CCT TGT ACC AGT CA | 157 | 50 | 98.56% | 0.987303 |
| EF2 | Elongation Factor 2 | COLO4_14020 | TGA TGG GTC AAG GTC TGA AA | ACT CGA GAA AGG CCA ACT GT | 131 | 50 | 91.71% | 0.992629 |
| ETIF3E | Eukaryotic translation initiation Factor 3E | COLO4_36514 | GGA GTT TGA GTG CAT TGT GG | GGT TCA AGG GTG AGG AGA AA | 129 | 50 | 93.51% | 0.993426 |
| GAPDH | Glyceraldehyde 3-phosphate dehydrogenase | COLO4_16834 | GTG AGA TCG GTA GGT TGG TG | CCT TGA CTT TGA GCT CGT GA | 159 | 52 | 92.7% | 0.998722 |
| PP2A | Catalytic subunit of protein phosphatase 2A | COLO4_36262 | TTG CTG CTC AGT TCA ACC AT | TTG GAG CAC TGA AGA CGG TA | 120 | 50 | 111.96% | 0.999715 |
| PTB | Polypyrimidine tract-binding protein homolog | COLO4_10326 | GGA ATG CCT TAG ATG GGA GA | ATA GTC CCT GCT CCG ATG AG | 134 | 50 | 93.75% | 0.998108 |
| UBC2 | Ubiquitin-conjugating enzyme E2 | COLO4_23034 | TCA GTC ATT GCT CTG TGA TCC | TCC AAC TCT GCT CGA CAA TC | 122 | 50 | 113.07% | 0.998592 |
| UBI1 | Ubiquitin 1 | COLO4_07010 | GTG CAG CAA AGG CTC ATT TA | CCT CTC AAA GCA AGC ACA AG | 110 | 50 | 93.38% | 0.995545 |
| ERF7A | Ethylene-responsive transcription factor (RAP2.3) | COLO4_13807 | AAA CCA CCG ACT CAT CAT CA | CTC CAA ACC AAG GAA CGA TT | 164 | 50 | 97.28% | 0.995791 |
| ERF7B | Hypoxia responsive ethylene-responsive transcription factor (HRE2) | COLO4_23306 | GCC TGC TCG TAA CAG TCA TC | TCT GCC TTT ACC GGT GTG TA | 177 | 53 | 99.75% | 0.998058 |
