# Supplementary material for: Identification and validation of reference genes for real-time quantitative RT-PCR analysis in jute
Source: BMC Mol Biol. 2019 Apr 29;20:13. doi: 10.1186/s12867-019-0130-2 (PMC6489354; doi:10.1186/s12867-019-0130-2)
Supplement: Supplementary file 2 — Additional file 2: Supplementary Figure. [file 12867_2019_130_MOESM2_ESM.docx]

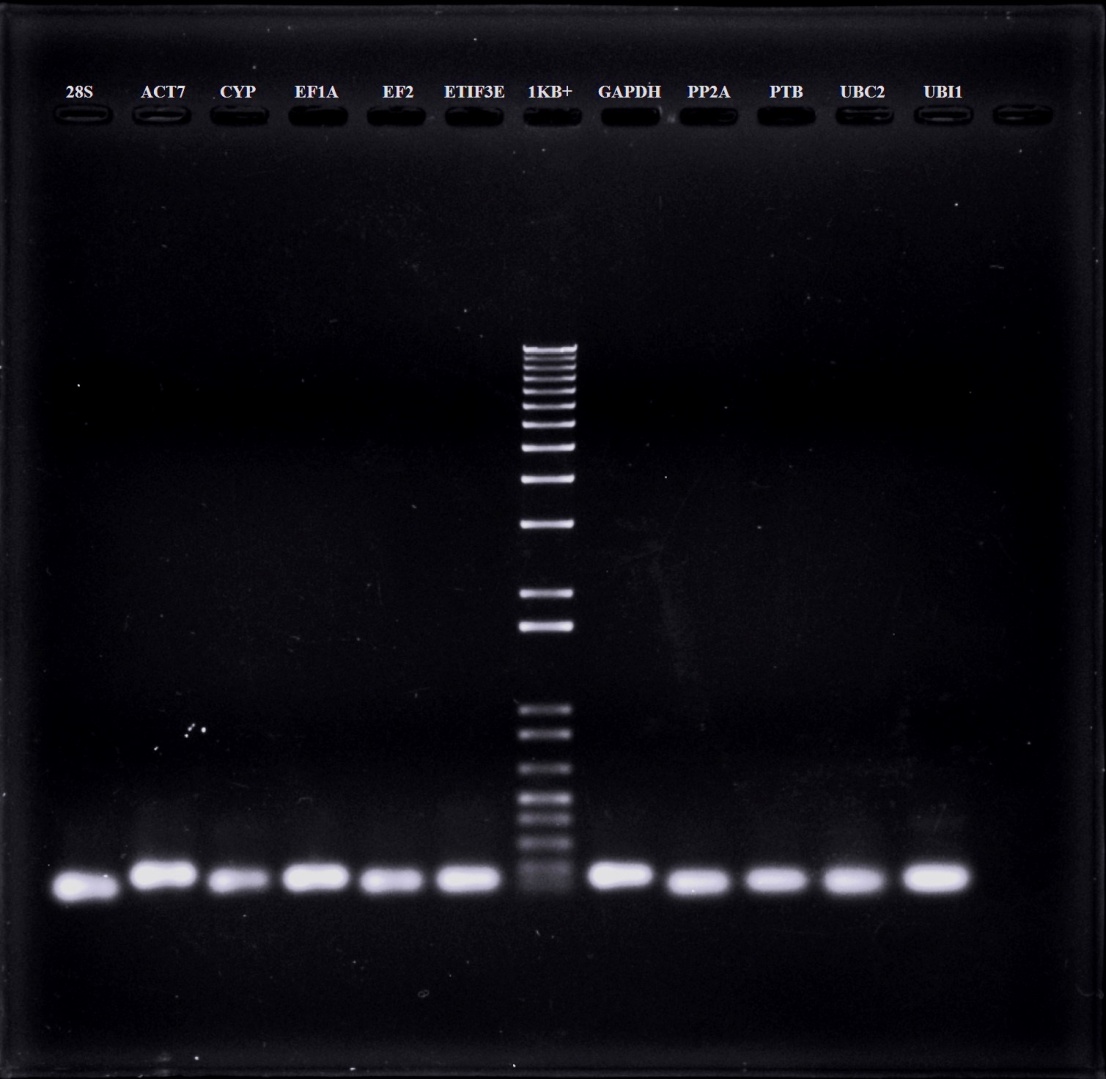


**Figure S1. Specificity of primers**. Agarose gel electrophoresis showing amplification specificity of the candidate housekeeping genes.


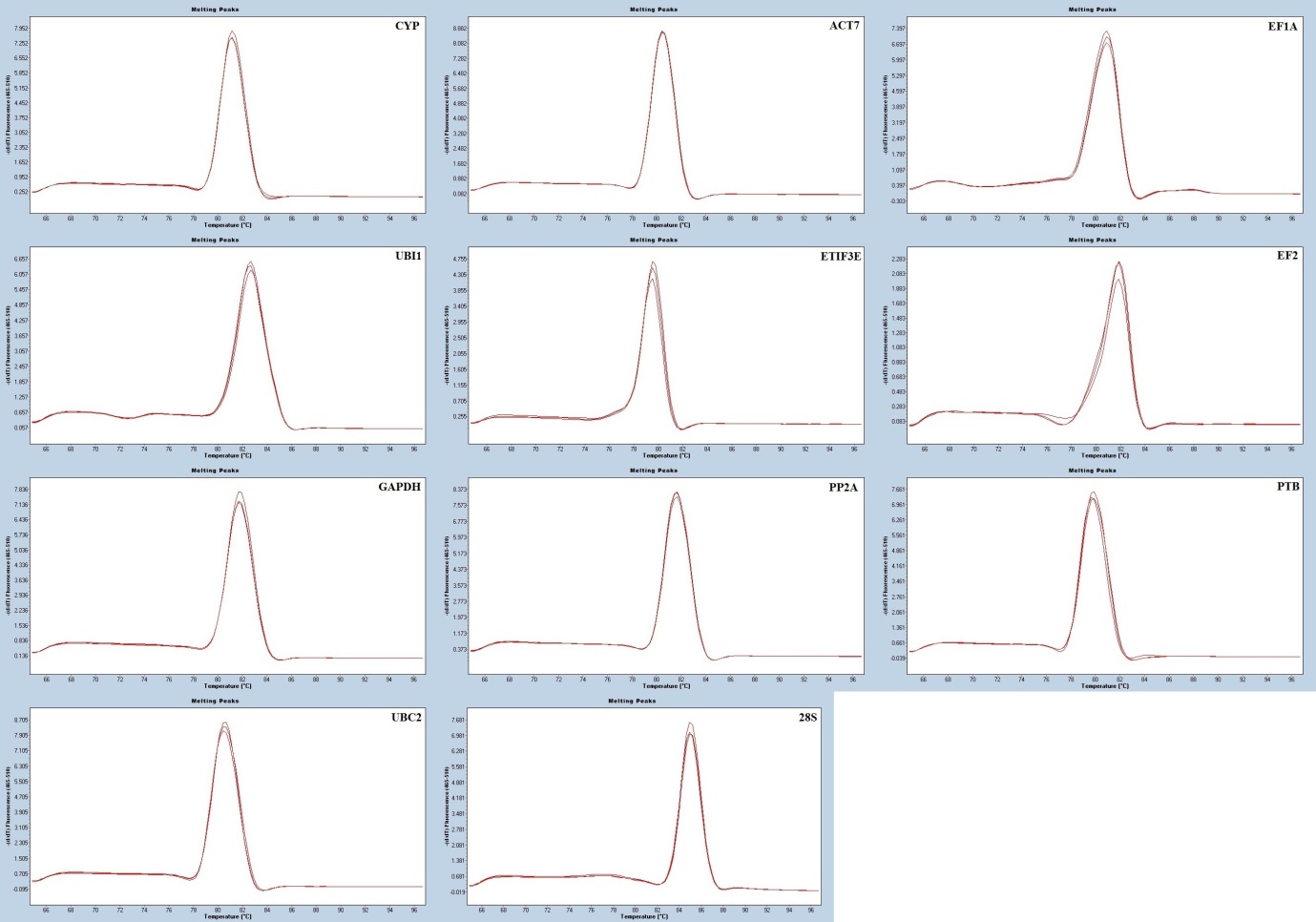


**Figure S2. Specificity of qRT-PCR amplification**. Dissociation curves of the 11 candidate housekeeping genes.with single peak after qRT-PCR reactions.


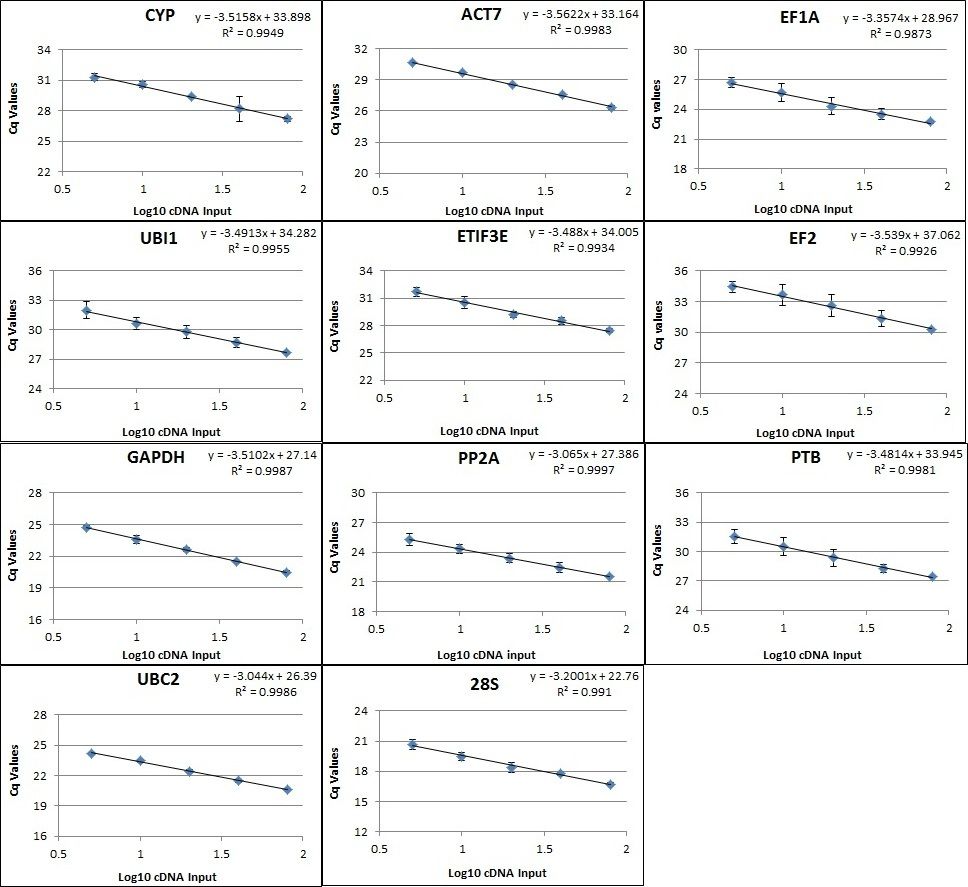


**Figure S3. Primer efficiency.** Standard curves obtained from real-time PCR for the 11 candidate housekeeping genes. The x-axis represents the copies of cDNA and y-axis represents the cycle threshold.
